# Isolation, expansion and neural differentiation of stem cells from human plucked hair: a further step towards autologous nerve recovery

**DOI:** 10.1007/s10616-015-9938-x

**Published:** 2015-12-24

**Authors:** Coen G. Gho, Timo Schomann, Simon C. de Groot, Johan H. M. Frijns, Marcelo N. Rivolta, Martino H. A. Neumann, Margriet A. Huisman

**Affiliations:** 1 Hair Science Institute, Maastricht, The Netherlands; 2 Department of Otorhinolaryngology and Head and Neck Surgery, Leiden University Medical Centre, Room J2-60 | Building 1, PO Box 9600, 2300 RC Leiden, The Netherlands; 3 Centre for Stem Cell Biology, The University of Sheffield, Sheffield, UK; 4 Department of Dermatology and Venereology, Erasmus University, Rotterdam, The Netherlands

**Keywords:** Hair follicle stem cell, Regeneration, Neural crest, Neuron, Glia, Cryopreservation

## Abstract

Stem cells from the adult hair follicle bulge can differentiate into neurons and glia, which is advantageous for the development of an autologous cell-based therapy for neurological diseases. Consequently, bulge stem cells from plucked hair may increase opportunities for personalized neuroregenerative therapy. Hairs were plucked from the scalps of healthy donors, and the bulges were cultured without prior tissue treatment. Shortly after outgrowth from the bulge, cellular protein expression was established immunohistochemically. The doubling time was calculated upon expansion, and the viability of expanded, cryopreserved cells was assessed after shear stress. The neuroglial differentiation potential was assessed from cryopreserved cells. Shortly after outgrowth, the cells were immunopositive for nestin, SLUG, AP-2α and SOX9, and negative for SOX10. Each bulge yielded approximately 1 × 10^4^ cells after three passages. Doubling time was 3.3 (±1.5) days. Cellular viability did not differ significantly from control cells after shear stress. The cells expressed class III β-tubulin (TUBB3) and synapsin-1 after 3 weeks of neuronal differentiation. Glial differentiation yielded KROX20- and MPZ-immunopositive cells after 2 weeks. We demonstrated that human hair follicle bulge-derived stem cells can be cultivated easily, expanded efficiently and kept frozen until needed. After cryopreservation, the cells were viable and displayed both neuronal and glial differentiation potential.

## Introduction

During the last decade, the interest in autologous stem cells has increased considerably, especially regarding the development of individualized therapies (Prasongchean and Ferretti 2012). However, the procurement of autologous somatic stem cells for human therapeutic purposes is still limited. In addition, somatic stem cell potency is restricted, and multipotent rather than pluripotent (Prasongchean and Ferretti 2012). Reprogramming somatic cells into induced pluripotent stem cells by the forced expression of certain genes is being explored but is controversial, since they are often tumorigenic and may initialize a T cell-dependent immune response in syngeneic recipients (Zhang et al. 2012; Zhao et al. 2011; Fu 2013).

For these reasons, the use of other types of autologous somatic stem cells is currently under investigation, such as bone marrow stem cells in a curative treatment for ischemic heart patients and cerebral infarction (Tian et al. 2014; Kasahara et al. 2013), already showing some promising results. One of the risks with this approach is that stem cells may follow their innate biological inclination irrespective of the tissue or organ into which they have been grafted. This was demonstrated by the finding that autologous bone marrow stem cells can produce extracellular matrix after engraftment into the brain (Grigoriadis et al. 2011; Snyder 2011). An alternative strategy could involve the use of neural crest-derived stem cells (NCSCs). They are appropriate for autologous cell-based therapy in many diseases, as they can be derived from adult tissue and can give rise to many cell types from ectodermal and mesodermal lineages. NCSCs from adult tissue (aNCSCs) are nononcogenic and possess a broad regenerative potential (Achilleos and Trainor 2012). In culture, aNCSCs retain their neural crest potential to differentiate into a variety of cells including adipocytes, chondrocytes, neurons, glia, osteocytes, and muscle cells (Achilleos and Trainor 2012). Minimally invasive, easily accessible sources for NCSCs are the olfactory sheath, palate, dental pulp and the hair follicle bulge (Achilleos and Trainor 2012). We consider the hair follicle to be the most easily accessible option (Huisman and Rivolta 2012). It has been reported that hair follicle bulge-derived NCSCs (HFBSCs) from human adults still possess neural crest characteristics such as multipotency (Mistriotis and Andreadis 2013; Sieber-Blum et al. 2004; Yu et al. 2010; Amoh et al. 2009a, b). This multipotency is of particular use in the area of neuroregeneration, given that hair follicle stem cells can promote the functional recovery of injured peripheral and central nerves (Amoh et al. 2008, 2009a, b; Hu et al. 2010). Hence, autologous HFBSCs are potentially suitable for therapeutic application in a broad range of neurological disorders such as ALS, Alzheimer’s disease and stroke. They also may be used in cell-based therapies for sensory neurological diseases such as those for ocular or inner ear regeneration (Liu et al. 2014; Huisman and Rivolta 2012). If HFBSCs could be harvested from plucked hairs, their practical utilization for autologous stem cell therapy would increase immensely. We therefore aimed to establish that:

1. Follicular stem cells, migrated out of the bulge from plucked hair follicles, are nestin-positive and possess a neural crest stem cell immunophenotype. Moreover, due to the cytotoxic nature of proteases, we intend to minimize the enzymatic treatments of tissue and cells (Aoshiba et al. 2001).
2. HFBSCs can be used for transplantation purposes, because they can be expanded easily and remain viable after cryopreservation and needle shear stress.
3. These stem cells are suitable as a source for future neural regenerative medicine in the patient, i.e. they can be stored frozen while keeping their neural and glial differentiation capacity.

## Materials and methods

### Specimen

**Fig. 1:**
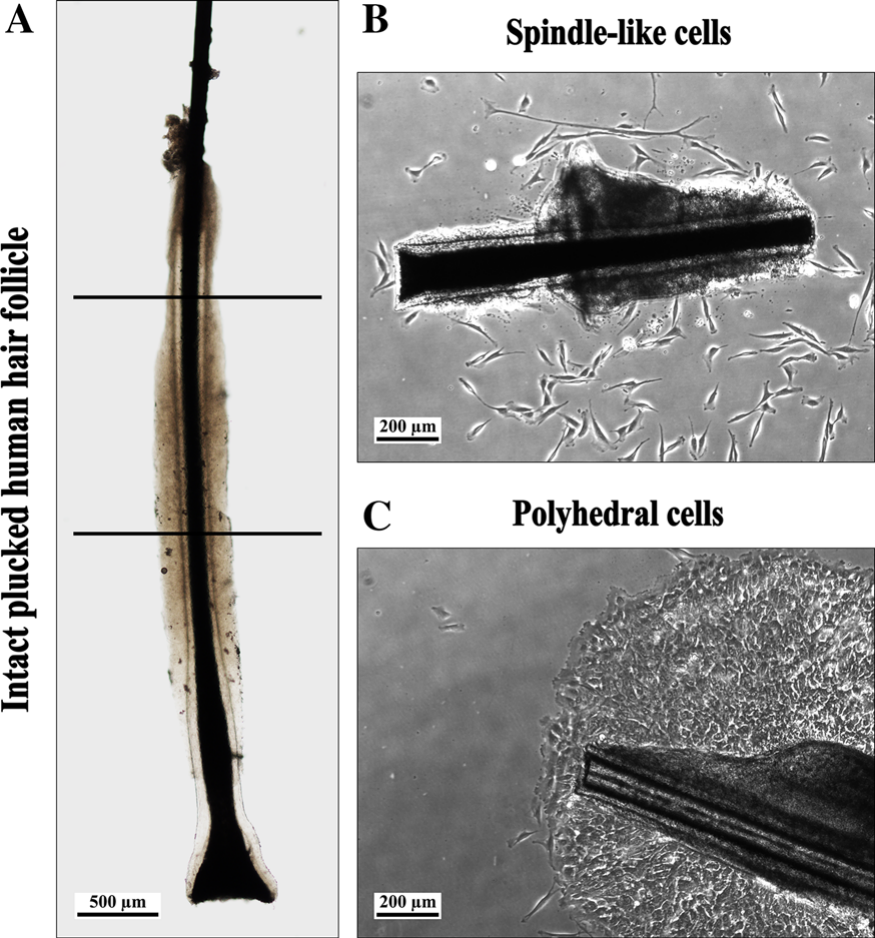
**a** Hair follicle with an intact inner and outer root sheath. Only the upper half of the follicle was used (between *lines*; *scale bar* 500 µm). **b** HF and cells with spindle-like morphology, at day 2 of outgrowth. The outer root sheath is curled (*scale bar* 200 µm). **c** HF and tightly clustered cells with an epithelial appearance (sheets of flattened polyhedral cells; *scale bar* 200 µm)

Plucked hairs from healthy donors were obtained from the occipital area of the scalp. The hairs were removed with depilation forceps. All human material was handled according to the Dutch Medical Treatment Agreement Act (Dutch Civil Code, Book 7, Section 7.7.5, article 7:467; http://www.dutchcivillaw.com/legislation/dcctitle7777.htm). Intact hair follicles (HFs) in the anagen phase were selected under a dissection microscope, and placed in DMEM/Ham’s F-12 1:1 (Biochrom AG, Berlin, Germany) containing 1 % GlutaMAX (100x; Life Technologies, Carlsbad, CA, USA) and 1 % Antibiotic Antimycotic Solution (100x; Sigma-Aldrich, St. Louis, MO, USA) (Fig. 1a). The HFs were processed the next morning.

### Isolation and cultivation of HFBSCs

Isolation of HF stem cells was according to Sieber-Blum et al. (2004) with minor changes. Briefly, connective tissue (if present) was removed from the HF and the bulge-containing area was dissected out just below the sebaceous gland and well above the bulb (Fig. 1a). Then, a longitudinal section along the tissue of the bulge was made, to cause the tissue to unfold. During these procedures, care has to be taken to avoid dehydration of the HF. Before the start of the culture, tissue culture 12-well plates (TPP; Trasadingen, Switzerland) were coated with poly-d-lysine (PDL; Sigma-Aldrich) diluted in sterile demi water (1:10) at 37 °C and 5 % CO_2_ for 1 h. Then the PDL solution was removed and the wells air-dried under sterile conditions. Prior to usage, the PDL matrix was rehydrated with basic growth medium (BGM, 37 °C, 30 min). BGM consisted of DMEM/Ham’s F-12 1:1, containing 1 % GlutaMAX, 1 % Antibiotic Antimycotic Solution, supplemented with 10 % fetal bovine serum Gold (FBS; Life Technologies), 2 % B-27 Supplement without vitamin A (50x; Life Technologies), 1 % N-2 MAX Media Supplement (100x; R&D Systems, Minneapolis, MN, USA), recombinant human Fibroblast Growth Factor-basic (rhFGF-basic; 20 ng/ml; R&D Systems), and recombinant human Epidermal Growth Factor (rhEGF; 20 ng/ml; R&D Systems). After rehydration, the BGM was poured out of the wells, and one HF-bulge was placed in each well. The HFs were carefully pressed on the bottom of the well using a forceps. Subsequently, three incubation periods in a small drop of medium allowed the HF to attach to the matrix. Incubation was done at 37 °C and 5 % CO_2_ for 75 min. If necessary, some medium was added. Finally, 500 µl of freshly prepared BGM was added cautiously. The primary culture was established by the outgrowth of HF stem cells from the bulge, usually at 8–10 days after the start of the culturing. After 1 week of culturing, a complete medium change was performed, followed by replacement of half of the medium every other day. Three to four days after the start of outgrowth, the HF bulge was removed and some of the cultures were fixed with 1 % formaldehyde in PBS (FA) for immunohistochemical analysis of neural crest markers.

### Expansion and cryopreservation

After removal of the bulge, cells were grown to 60–70 % confluence and enzymatically detached using pre-warmed 0.05 % trypsin–EDTA (Life Technologies) at 37 °C for precisely 2 min. Trypsinization was stopped by the addition of DMEM/HAM’s F-12 1:1 supplemented with 10 % FBS. The cells were centrifuged at 280×*g* for 10 min, and the cell pellet was suspended in 1 ml BGM. After cell counting (Logos Biosystems, Anyang-City, Korea), the cells were seeded at expansion density (2.5 × 10^3^ cells per cm^2^) in a PDL-coated dish and allowed to expand until 60–70 % confluence. In general, cells were passaged three to four times. Each period of time prior to passaging was about 1 week. Doubling times were calculated at passages 2 and 3, using the site: Roth V. 2006 Doubling Time Computing, Available from: http://www.doubling-time.com/compute.php (Kim et al. 2011).

In addition, a portion of the cells was frozen at −80 °C at a concentration of 1 × 10^6^ cells/ml in 90 % FBS with 10 % dimethyl sulfoxide (Sigma-Aldrich). After storage and thawing, the cells were suspended in 5 ml BGM, centrifuged, collected, suspended in BGM, carefully triturated, seeded at expansion density, and cultured at 37 °C and 5 % CO_2_.

### Simulation of the transplantation procedure: ejection of cells

After cryopreservation, cells were cultured at 37 °C and 5 % CO_2_. After 1 week cells were enzymatically detached and centrifuged at 280×*g* for 10 min. They were suspended at a density of ~4.0 × 10^6^ cells/ml in BGM medium and carefully triturated. Subsequently, 10 μl of the cell suspension was loaded into a 100 μl syringe with a 30 gauge needle and injected into a 1 ml Eppendorf tube using a programmable syringe pump (Prosense, Oosterhout, The Netherlands); settings: diameter 4.699 mm—rate 0.5 ml/min. Both cultured and cryopreserved cells were subjected to shear stress.

Viability was assessed using the trypan blue test. Trypan blue staining is based on the principle that live cells possess intact cell membranes that exclude the dye, whereas dead cells do not (Strober 2001). A 1:1 dilution of cell suspension and 0.4 % trypan blue was incubated for 2 min at room temperature. Next, the stained cells were counted using a Neubauer haemocytometer chamber and calculated using the following formula: vital cell rate (%) = number of vital cells/(number of vital cells + number of dead cells) × 100 %. Cells that had been cultured but not injected served as controls.

### Statistical analysis

The paired, two-tailed Student’s *t* test was used to estimate the difference between the control and injected cells. The unpaired, two-tailed Student’s *t* test was used to estimate the difference between control cells and both cryopreserved and injected cryopreserved cells.

### Neural differentiation of HFBSCs

Following outgrowth, expansion, and cryopreservation of HFBSCs, 2.5 × 10^5^ cells in 500 µl of BGM were seeded per well of a 12-well plate. The cells were seeded via the side into PDL-coated wells containing PDL-coated cover glasses (Thermo Scientific, Waltham, MA, USA). It was essential in all the described procedures to prevent the cover glass sticking to the bottom of the well. Prior to PDL coating, the cover glasses were etched in 85 % phosphoric acid (Merck Millipore, Darmstadt, Germany) for 12 h (Beaudoin III et al. 2012). Subsequently, acid-treated cover glasses were rinsed extensively in ultrapure water and subjected to a graded series of 70, 90, and 96 % ethanol. Cover glasses were stored in 96 % ethanol. The 12-well plates and etched cover glasses were coated separately with PDL as described previously. After seeding, the cells were cultured at 37 °C and 5 % CO_2_, while their settlement underneath the cover glass was observed daily. When an appropriate density was achieved, i.e., 5 to 10 cells in one field of view (FOV, 10× magnification, an area of ~3.5 mm^2^), differentiation was induced by removal of 250 µl medium and replacement with 300 µl cAMP-containing induction medium (IM) (Jarmalavičiūtė et al. 2013). IM consisted of DMEM/Ham’s F-12 1:1 supplemented with 1.5 mM cAMP (Sigma-Aldrich), 1 % glutamax (Life Technologies), 10 ng/ml NGF, 10 ng/ml GDNF, 10 ng/ml BDNF (all from R&D Systems) and 2 % B27 + VitA (Life Technologies). If the appropriate density was not achieved, half of the medium was replaced with fresh BGM every other day. After IM was added, the cultures were allowed to differentiate for at least 60 h without disturbance due to opening of the incubator or observation of the cells. Subsequently, 250 µl medium was removed and again substituted with 300 µl IM. Thereafter, the medium was replenished with IM every other day. Cultures were observed for morphological changes on a daily basis. If no neuronal morphologies appeared after 7 days of differentiation, the culture underwent another period without disturbance in IM, and the above-mentioned differentiation procedure was followed again. After differentiation for 7–14 days, the cover glass was carefully removed, because cells were not only attached to the bottom of the well but sometimes also to the underside of the cover glass. The cells on the bottom of the well were fixed in 1 % FA for 15 min and processed for immunohistochemistry. Fixed cells were stored at 4 °C for a maximum period of 2 weeks.

### Glial differentiation of HFBSCs

After expansion, a volume of 500 µl of BGM (without FBS) containing 1 × 10^5^ cells was pipetted into each well of a 12-well plate. The cells were seeded via the side into uncoated wells which contained PDL-coated cover glasses. During culture, the cover glass should not stick to the bottom of the well. On the next day, half of the medium was replaced with serum-free BGM (Pacey et al. 2006). Cells were observed every other day to follow settlement and changes in morphology. The medium was exchanged with serum-free medium every other day until an average density of 10–20 cells per FOV underneath the cover glass was reached. Then half of the medium was replaced by IM. The cells underneath the cover glass usually adopt glial-like morphologies after 3–4 days of induction. After induction, the medium was exchanged once a week with IM. Cultures were maintained until networks of cells were observed. Because the networks were attached to the underside of the cover glass, the cover glasses were removed and placed upside down in another well of a 12-well dish. The cells were fixed in 1 % FA for 15 min.

### Immunohistochemistry

Prior to immunohistochemistry, the cells were washed with 0.05 % Tween-20 in PBS for 5 min and permeabilized in 0.1 % Triton X-100 in PBS for 10 min. Cells were treated with blocking solution consisting of 5 % non-immune serum in 0.05 % Tween-20 in PBS for 30 min. Afterwards, the cells were incubated with the primary antibodies in blocking solution at 4 °C overnight (Petersen and Pedersen 2013). The primary antibodies used were: anti-Nestin (1:500, Biosensis, Thebarton, South Australia), anti-SLUG (1:125, Abcam (Cambridge, U.K.) ab 27568), anti-AP-2α (1:100, Santa Cruz Biotechnology (Santa Cruz, CA, USA) sc-53164), anti-SOX9 (1:500, Millipore (Billerica, MA, USA) AB5535), anti-SOX10 (1:200, Santa Cruz sc-17342), anti-β-III-tubulin (1:200, Abcam ab18207), anti-synapsin-1 (1:200, Abcam ab8), anti-myelin protein zero (MPZ; 1:200, Neuromics (Minneapolis, MN, USA) Ch23009), and anti-Krox20 (1:100, Covance, New York, NY, USA). The secondary fluorochrome-conjugated antibodies were diluted 1:500 in blocking buffer, and the cells were incubated at room temperature for 1 h. The secondary antibodies were conjugated with either Alexa Fluor 488 or Alexa Fluor 555 (Life Technologies). Nuclear counterstaining was performed with 1:1000 DAPI (Life Technologies, D3571) in PBS. The cells were covered with Vectashield (Vector Laboratories, Burlingame, CA, USA). Omission of the primary antibody served as a control for false cross-reactivity of the secondary antibody. Pertinent positive cell or tissue controls were used: RT4-D6P2T, a rat Schwann cell line (ATCC, Manassas, VA, USA) for anti-nestin, -SOX9, -Krox20, and -MPZ, the Melan-Ink4a cell line (Wellcome Trust Functional Genomics Cell Bank, London, UK) for anti-SOX10 and -β-III-tubulin (Locher et al. 2013), the SKBR3-breast cancer cell line for anti-SLUG and Ap-2α, and mouse brain for anti-synapsin-1.

Fluorescence imaging was performed using fluorescence microscopy (Olympus IX70) in combination with the LAS AF microscope software (Version 1.9.0 build 1633, Leica Microsystems). The data were corrected for background staining and normalized using the quantification method of the software. Only those immunostainings showing a peak maximum at emission of at least two times higher than the background were considered to be significantly positive. Pictures were processed using Adobe Photoshop CS6 Extended (Version: 13.0 x64, Adobe Systems Incorporated, San Jose, CA, USA).

## Results

### Isolation and cultivation of HFBSCs

In our experience, more than 60 % of the bulges remained attached and produced cellular outgrowth (Table 1). However, outgrowth did not always yield the desired phenotype of HFBSCs with spindle-like morphology (Fig. 1b); tightly clustered cells with an epithelial appearance were also seen (sheets of flattened polyhedral cells, Fig. 1c). Both cell phenotypes emigrated from bulge explants at 8–10 days of culture. Based on morphology and immunohistochemistry (results not shown), the flattened cells were identified as keratinocytes. Therefore, cultures containing those cells were discarded (Table 1).

**Table 1:** Outgrowth, adhesion and morphology of cells from the HF bulge explant

Cultures	HFs planted (n)	Cultures with outgrowth (%)	Cultures with polyhedral cell outgrowth (%)	Cultures with spindle-like cell outgrowth (%)
905E1P0	12	58	33	57
906E1P0	12	58	57	43
916E1P0	12	67	37	63
932E1P0	12	67	100	0
934E1P0	30	67	75	25
Average	–	63	Discarded	34

### In vitro NCSC-characteristic protein expression profile

**Fig. 2:**
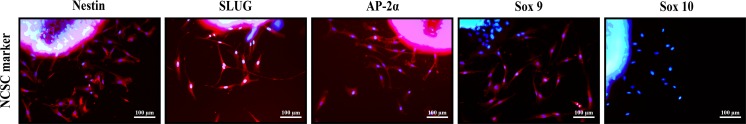
HFBSCs at day 2 of outgrowth. The NCSC markers nestin (*red*), SLUG (*red*), AP-2α (*red*) and SOX9 (*red*) are positive. SOX10 (*red*) is negative. Nuclei are stained with DAPI (*blue*; *scale bar* 100 µm)

The majority of HFBSCs express the neural crest cell markers SOX9, SLUG, and AP-2α as determined by indirect immunohistochemistry (Meulemans and Bronner-Fraser 2004). SOX10 expression was below the level of detection. The neural progenitor cell marker nestin was present in all cells (Fig. 2a–e).

### Expansion and cryopreservation

The selected primary (P0) cultures reached 60–70 % confluence after 15–19 days. The P1 cultures reached 80 % confluence after 1 week and those from P2, 1 week later. The total yield of cells per HF was approximately 1 × 10^4^. The mean doubling time, calculated from the P2 and P3 cultures, was 3.3 (±1.5) days (Table 2).

**Table 2:** Doubling times of HFBSCs after passaging

Cultures	Cells at start of culture (n)	Cells at end of culture (n)	Doubling time (days)
905P1 to P2	199,000	1,872,000	2.8
916P1 to P2	328,000	2,128,000	5.6
934P1 to P2	60,000	1,000,000	2.7
1011P2 to P3	350,000	3,800,000	2.9
1012P1 to P3	350,000	4,400,000	2.7
Avg ± SD			3.3 ± 1.5

### Simulation of the transplantation procedure: ejection of cells

Shear stress caused by injection through a syringe needle did not change the viability of the cells significantly, whether the cells were freshly cultured or frozen (*p* = 0.2401 and *p* = 0.6306, respectively). However, a small but significant difference in viability was found between cells which were only cultured and those which were cryopreserved (*p* = 0.009). Nevertheless, after cryopreservation, 82.2 % ± 2.33 % of the cells were still viable. These results show that HFBSCs can be expanded and kept frozen until needed.

### Neural differentiation

**Fig. 3:**
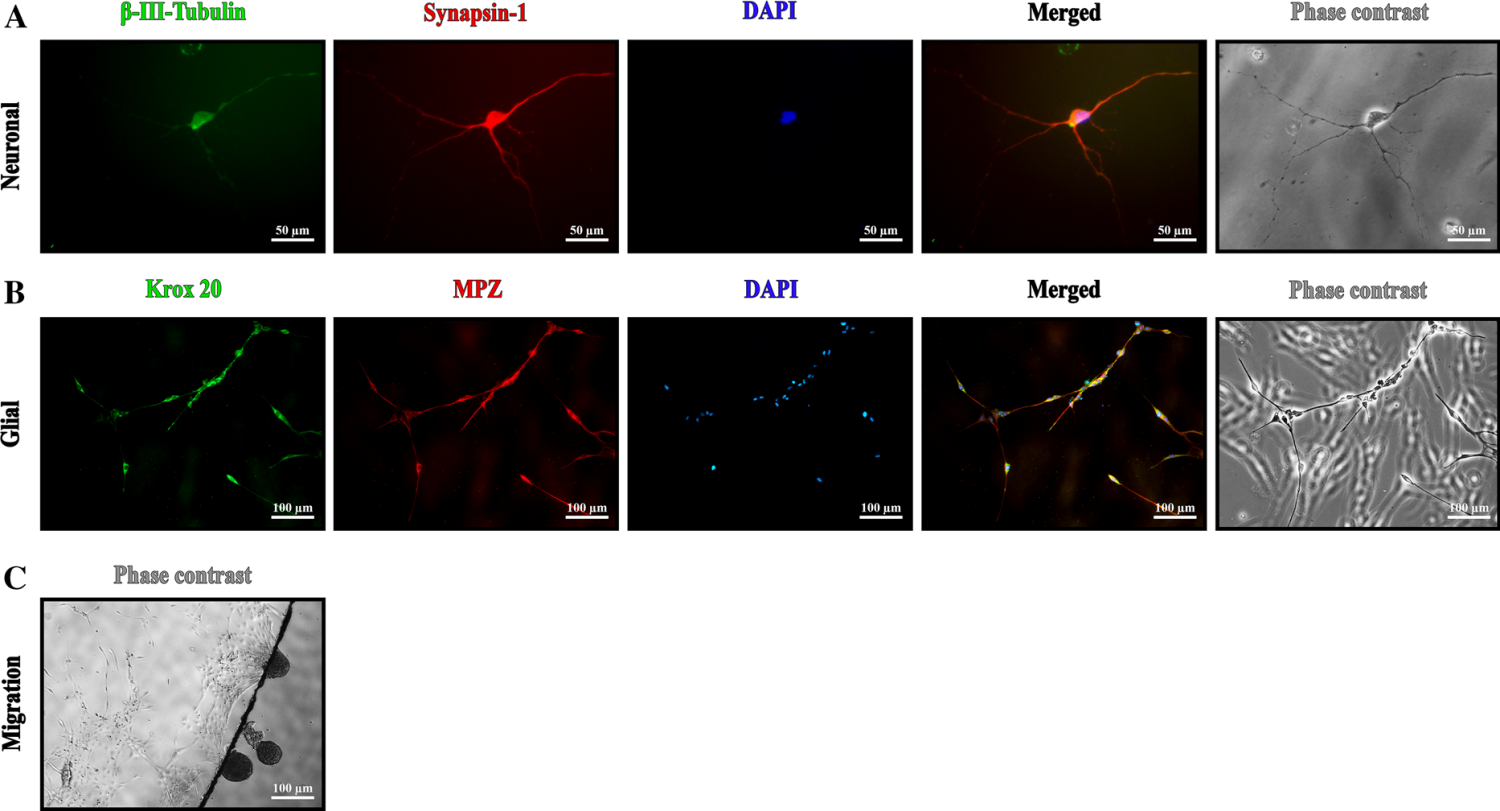
**a** Representative HFBSCs 14 days after neuronal induction. Cells at the bottom of the well stained positive for βIII-Tubulin (*green*) as well as synapsin-1 (*red*). The cell nucleus is stained with DAPI (*blue*). Merged image reveals overlay of βIII-Tubulin (*green*) as well as synapsin-1 (*red*) localization. The phase contrast image shows differences in long dendritic projections and thicker axon (*scale bar* 50 µm). **b** HFBSCs 15 days after glial induction. Glial cells were at the underside of the cover glass, therefore cells on the top of the glass are also faintly visible, but out of focus. Cells stained positive for the glial markers Krox20 (*green*) and MPZ (*red*). Nuclei are stained with DAPI (*blue*). Cells with a *yellow* *color* in the merged image co-express MPZ (*red*) and Krox20 (*green*). The phase contrast image depicts spindle-shaped morphologies of the glial cells (*scale bar* 100 µm). **c** Migration of glial cells underneath the cover glass

For neural differentiation, we used the principle of the ‘sandwich method’. The rationale for this method is, that neuronal survival is improved if the cells are grown on a substrate-coated surface and covered by a cover glass (Brewer and Cotman 1989). In general, a density of about 8 cells per FOV (~3.5 mm^2^) underneath the cover glass gave the highest number of surviving neuron-like cells during neuronal induction. A considerable number of cells on top of the cover glass could not endure neuronal induction and died, resulting in cellular debris. Too much of this debris appeared to be destructive to neurons, because it covered the developing neuron-like cells and their projections, vacuoles were formed and the neurons subsequently deteriorated. Under the condition that cells were not covered by cellular debris, different neuron-like cells with elongated, branched projections developed over time. These projections could grow to a length ranging between 100 and 500 µm (Fig. 3a). Neuron-like cells were found mainly on the bottom of the well.

### Glial differentiation

After seeding many cells attached within a few minutes to the PDL matrix, but a considerable number of HFBSCs remained motile. A considerable number of these cells migrated underneath the cover glass, sometimes in globular cell aggregates (Pacey et al. 2006). These cells were mainly bipolar, with a distinct bulbous soma and small projections. After 2 or 3 days, most of these cells showed premature glial-like morphologies. A few days later, their soma became spherical and the first projections appeared, all very different in length. These glia-like cells quickly formed spacious, structured networks. With further culturing, the cells and networks did not alter noticeably.

### Immunohistochemistry

In general, many cells were β-III-tubulin-positive early during neural induction, and synapsin-1-positivity was detectable a few days later (Fig. 3b–d). Glial induction yielded cells which were β-III-tubulin-negative, but positive for MPZ and Krox20 (Fig. 3b–d).

## Discussion

We isolated, expanded and cryopreserved nestin-positive stem cells, derived from the bulge area from plucked human hairs. We showed that these stem cells, which are also positive for the neural crest markers SOX9, SLUG and AP-2α, are suitable for transplantation purposes because they easily survive cryopreservation and needle shear stress, while conserving their neuronal differentiation capacities.

The technique used to harvest the HF is virtually painless and allows the collection of hundreds of HF per patient. The culture technique to expand the cells is simple and straightforward and usually yields 1 × 10^4^ cells per HF. Only three to four passages are required for this high yield, so cellular senescence is not an issue. Moreover, cellular damage by frequent protease treatments is restricted to a minimum. We do not consider the series of conditions mentioned in this report as imperative protocols, prerequisite to stimulating these cells towards differentiation into neurons and glia cells. There are some critical points in the procedures, however, such as the selection of intact hair follicles, rejection of cultures with undesired cell outgrowth, and the sandwich differentiation culture method. Rigorous rejection of cultures based on critical observations and knowledge of contaminating cell types allowed us to perform a “selection at the gate” of the desired cell type. Obviously, the group of cells which are most prominently present in the hair follicle are keratinocytes. These cultures were immediately discarded. Other cells present in the hair follicle are fibroblasts, which possess in the migrating phase—like many other cells—a spindle-like morphology. However, cultures containing fibroblasts will soon be overgrown by this contaminating cell type, for the doubling time of human dermal fibroblasts is in general 24 h, while the hair follicle bulge stem cells have a doubling time of an average of 3.3 days (see also “Results”, section Expansion and cryopreservation). Fibroblasts will thus overgrow the other cells and form arrays of cells oriented in a curvilinear pattern. These cultures were also discarded. Other cells, which have been reported to be present in the hair follicle are melanocytes, cells from the peripheral nerve ends and some muscle cells (Locher et al. 2013). Melanocytes, which in the hair follicle have a similar morphology as the bulge cells (bipolar), do not grow under the culture conditions reported here: they need medium with a relatively low pH, generated by 10 % CO_2_. We did not consider Schwann cells from the peripheral nerve endings as contaminating, for they are also neural crest-derived. Muscle cells are sometimes present in mouse vibrissae cultures, but we have never demonstrated them in human hair follicle cultures (using αSMA staining).

Using the sandwich differentiation method, it was possible to vary the culture circumstances in such a way that a different neural cell phenotype was achieved. It is known that a combination of serum deprivation, cell density and substrate can direct neural stem cells to develop towards a neuronal or glial phenotype (Hung and Young 2006). In the neuronal differentiation protocol, the surface of the cover glass was soon covered with many cells, due to the relatively high seeding density and proliferative stimulus of FBS. This apparently prevented the attachment of many cells. Thus, different cell types remained floating and finally found a habitat underneath the cover glass. Some of these cells showed a fibroblast-like morphology, while others were neuron-like cells with a small, shining soma and two or more thin projections. In time, the fibroblast-like cells disappeared; they apparently did not survive the differentiation medium. However, there was obviously contact between the small neuron-like cells and the fibroblasts, suggesting paracrine interactions which may stimulate neural differentiation (Moore et al. 2012). We assume that, because of the decrease in cell density, neuron-like cells develop long and complex branches in order to seek contact with other cells. It is conceivable that the micro-environment underneath the cover glass, such as a low oxygen pressure and the build-up of autocrine factors, might facilitate neuroglial differentiation and survival of HFBSCs (Brewer and Cotman 1989). Interestingly, in both protocols, cells with a shining soma and thin projections preferred migrating underneath the cover glass while other cells apparently preferred to settle on top. This cell type-specific affinity to seek a place in the narrow space between the cover glass and the bottom of the well, or on top of the cover glass, also occurred when HFBSCs were seeded underneath the cover glass (results not shown).

We are convinced that the repertoire of nestin-positive stem cells from the human HF bulge will be of benefit for many different autologous cell-based therapies. Still, there seems to be a difference of opinion about the precise localization of these cells. Clewes et al. (2011) described nestin-positive cells migrating from the HF bulge in culture, whereas Amoh et al. (2009a, b) found nestin-positive cells immediately below the sebaceous glands just above the bulge area, i.e., in the isthmus region. In our experience, it is nearly impossible to distinguish the bulge area from the isthmus region in human HFs (Fig. 1a). We therefore assume that the nestin-positive cells in our cultures and those described by Clewes et al. (2011) do not differ from those described by Amoh et al. (2009a, b), as was also previously suggested by Djian-Zaouche et al. (2012). Remarkable positive results of neural regeneration and functional improvement have been obtained in animal models of brain, spinal cord, and nerve injury therapy using stem cells derived from hair follicles (Amoh et al. 2008, 2009a, b; Hu et al. 2010), giving hope for patients suffering from neurodegenerative diseases. Another point of interest, however, is the predisposition of these HFBSCs to repair cranial bone, nerves and tissue of the head, e.g. in patients with severely burnt faces. These patients often have enough remaining hair to perform hair transplantations and to harvest stem cells, which may open possibilities for complete autologous facial repair. This holds true for autologous stem cell therapy in general, as experience with the culturing of stem cells in autologous serum is increasing (Duggal and Brinchmann 2011).

## Conclusion

We have demonstrated that stem cells from HFs, plucked from human scalps, display a NCSC immune profile and can be cultivated, expanded, and kept frozen until needed. We also showed that differentiation of these stem cells into neurons and glial cells is feasible. The technique used to harvest the HFs is almost painless and allows the collection of hundreds of HFs per patient, which is highly advantageous, making these cells very attractive for autologous transplantation and treatment models for a variety of disorders.
